# Risk assessment for *Brucella suis* biovar 2 in Danish pigs

**DOI:** 10.1186/s40813-025-00471-4

**Published:** 2025-11-17

**Authors:** Lis Alban, Annette Dresling, Maybritt Kiel Poulsen

**Affiliations:** 1 Department of Food Safety and Veterinary Issues, Danish Agriculture & Food Council, Copenhagen, Denmark; 2 Department of Veterinary and Animal Sciences, University of Copenhagen, Frederiksberg, Denmark

**Keywords:** Brucellosis, Herd health, Finishing pigs, meat inspection, Risk analysis, Risk-based inspection

## Abstract

**Background:**

In Denmark, the number of uncastrated finishing pigs is on the increase, due to a wish to improve animal welfare and productivity. According to the EU legislation, all parts of a slaughter animal must be subjected to official inspection, with the penis as an exception if already discarded. The question is what the risk associated with not detecting abnormal testicles is for people working with pigs and consumers eating pig meat. To address this, we undertook a semi-quantitative risk assessment following WOAH guidelines, assuming two different transmission pathways (contact/meat-borne) and two different pig populations (indoor/outdoor) using Denmark as a case.

**Results:**

*Brucella* spp. was identified as the only relevant public health hazard. Brucellosis is a notifiable zoonotic infection, among others resulting in enlarged testicles in infected boars. Denmark is officially free from *B. abortus* and *B. melitensis*, whereas *B. suis* biovar 2 has been detected on a few occasions during the last 50 years. No human cases have ever been detected in Denmark, and only few cases are found elsewhere. Pig meat is not ascribed to transmission of infection unless the meat comes from acutely infected animals.

**Conclusion:**

The human health risk related to *B. suis* biovar 2 was assessed as low in case of contact with outdoor-reared pigs and negligible for the meat-borne route and contact to indoor raised pigs. Therefore, pig producers and veterinarians should focus on outdoor-raised pig production and react immediately upon presence of relevant symptoms such as enlarged testicles or abortions.

**Supplementary Information:**

The online version contains supplementary material available at 10.1186/s40813-025-00471-4.

## Introduction

For decades, the male pigs from most of the world have been castrated to avoid boar taint; a natural odour that is present in the meat from intact male pigs. Some consumers perceive boar taint negatively, particularly in relation to consumption of heat-treated meat. This implies that boar taint is perceived as a quality failure [1]. To avoid subsequent development of boar taint, most male piglets have been castrated at an age of 3–5 days. However, castration is associated with pain and discomfort for the male piglet even though anaesthesia and analgesics are used [2], and it can, in some cases, cause higher mortality in male piglets compared to female piglets [3]. Additionally, castrated piglets have a higher food conversion ratio than non-castrated male piglets, which implies poorer productivity [4]. Hence, pig producers are interested in avoiding surgical castration of male pigs. All these issues have been a driver for work on how to abolish castration, although there is no easy solution to the challenges [2]. Development of an electronic nose to detect intact male carcasses with boar taint is in the pipeline, which will help in detecting carcasses with boar taint [5]. Moreover, immuno-castration using the veterinary immunological product Improvac^®^ (Zoetis Inc., Parsippany, New Jersey, USA) seems to be a feasible alternative because it prevents boar taint [6].

Denmark is a pig-producing country, with a population of 6 million people, and around 30 million pigs born in a year. Most pigs are raised indoors at farms that are officially considered as applying controlled housing conditions, implying a very high level of biosecurity [7]. Since 2023, Danish pig producers are delivering intact male or immuno-castrated finishing pigs for slaughter in large numbers. According to the EU Commission Regulation 2019/627, the carcass and associated organs, should be subjected to a post-mortem (PM) inspection [8]. The penis can be removed before the PM inspection by the food business operator and will in that case not be intended for human consumption but disposed of as an animal byproduct. The remaining parts of a slaughtered animal must remain identifiable as belonging to the corresponding carcass until the PM inspection is completed by the competent authority. If blood or other edible by-products, including the testicles, from multiple slaughtered animals are placed in one container as a batch before the inspection is finished, the entire content is declared as unfit for human consumption if a carcass of one of those slaughtered animals from the batch is condemned [9].

One Danish abattoir had slaughtered intact, male finishing pigs for more than 25 years before the plant was closed in 2024. The experience gained was that it was important to avoid contamination of the carcass with semen, testicle tissue and faecal material. To ensure this, the plant removed the testicles after singeing and black scraping/polishing, but before loosening of the rectum and dividing the carcass. However, this made it a challenge to present the testicles for PM inspection together with the carcass and organs from the corresponding animal.

Abattoirs can be very different in their set-up and organisation. In Denmark, robot technology is used extensively. The available space and types of robots used determine what is possible regarding inspection and where inspection is performed. Some abattoirs may find it feasible to keep the testicles on the carcass until after the carcass has been split, enabling easy and traditional PM inspection of the testicles, whereas other abattoirs would find that challenging.

However, before going into any discussion of how and where to inspect the testicles, it would make sense to identify the human health hazards that could be associated with not detecting abnormal testicles. Denmark is officially free from most notifiable livestock infections, but it is not free from all species of the genus *Brucella* [10], and the first hazard related to testicles of increased size that arose from a discussion between the authors was *Brucella. Brucella* are Gram-negative coccobacilli that cause the infection brucellosis, known for causing swelling of testicles and abortions. There are several species of *Brucella*, with the three most important being *B. abortus* (mainly in cattle and bison), *B. melitensis* (mainly in sheep and goats) and *B. suis* (the main animal species involved depends on the biovar involved). Due to their societal significance, these hazards are listed in the Terrestrial Animal Health Code developed by the Animal Organisation on Animal Health (WOAH) [11]. *Brucella* are zoonotic, implying that they can be transmitted between several animal species and humans. Therefore, in the case of suspected animal brucellosis, a veterinarian must be called, and if the suspicion cannot be rejected, the veterinarian must immediately report the finding to the competent authority, which will notify the WOAH [11].

According to the EU Commission Decision 2003/467/EC10, Denmark is recognised as officially free of *B. abortus* in cattle herds, and the last case was observed in 1962. Since 1995, Denmark has also been recognized by the EU as officially free of *B. melitensis*. In fact, *B. melitensis* has never been detected in Denmark. However, *Brucella suis* biovar 2 has been observed in Denmark on a few occasions [10].

*Brucella suis* is divided into five biovars that differ in terms of geographical distribution, hosts and zoonotic potential [12]. In the USA, pigs and wild boar (*Sus scrofa*) are reservoir hosts for biovars 1 and 3, whereas biovar 4 is found in caribou and reindeer (*Rangifer tarandus*) in Artic regions, including Alaska [13], where it can spillover to, e.g., the musk ox (*Ovibos moschatus*) [14]. In Europe, *B. suis* biovar 2 is the most common biovar and can be found in wild boar and the European brown hare (*Lepus europaeus)*, although biovars 1 and 3 can also occur [12]. In Australia, biovar 1 circulates in feral pigs [15], and finally, biovar 5 is found in rodents in the Caucasus area [16, 17]. The zoonotic potential varies between the biovars, with biovar 1 having the highest potential and biovar 2 the lowest [12].

According to the EU Regulation 2018/1882, *Brucella suis* in pigs is categorised as a List D disease, which means a disease for which measures are needed to prevent it from spreading on account of its entry into the European Union (EU) or movements between Member States. This is in contrast with *B. suis* in bovines, sheep or goats, which is considered a List B disease, which means a listed disease which must be controlled in all EU Member States with the goal of eradicating it throughout the Union [18]. This way of looking at *B. suis* biovar 2 in pigs mirrors the outcome of a risk assessment of *B. suis* undertaken by European Food Safety Authority (EFSA) in 2009. Before 2009, there was no requirement for surveillance for *B. suis* in domestic pigs or the wild fauna, and therefore, there were no systematic data regarding the occurrence of *B. suis*. EFSA assessed that the occurrence was mainly sporadic, except for special geographical areas, where the production form resulted in endemic infection [12].

In 2020, a new EU Regulation 2020/688 came into force, introducing monitoring requirements in connection with the movement of animals within the EU [19]. The new regulation was based on EFSA’s risk assessment and its conclusions. According to Article 19 in the Regulation, focus of the monitoring is outdoor-raised pigs. According to Annex 3 in the Regulation, the minimum requirement for monitoring includes an annual animal health visit by a veterinarian, which is carried out for many other reasons than *Brucella*. If pigs are kept for breeding purposes, there must also be an annual immunological examination of the herd. However, the annual animal health visit and the immunological examination are not necessary if the competent authority considers the risk of infection with *B. abortus*, *B. melitensis* and *B. suis* to be negligible in the Member State or zone and if the following conditions are fulfilled:

- No infection with *B. abortus*, *B. melitensis* and *B. suis* has been reported in the population of reared pigs in the last five years,
- No infection with *B. abortus*, *B. melitensis* and *B. suis* has been reported in the wild animal population of listed species in the last five years, and during that time, wild boars have been part of the target animal population monitoring,
- The Member State or zone therein is free from infection with *B. abortus*, *B. melitensis* and *B. suis* in respect of the bovine, ovine and caprine populations.

If infection with *B. abortus*, *B. melitensis* or *B. suis* has been reported in pigs in a herd, those animals can only be moved to another EU Member State if all pigs in the herd have undergone two consecutive tests with negative results. The first test must be carried out on samples taken at least three months after the removal of the infected animals from the herd. The second test must be carried out on samples taken at least six months and at most 12 months after the first test [19].

According to EFSA, the transmission of infection from wild fauna to outdoor herds primarily depends on, (1) the presence of infected wild boars and hares in the area, and (2) the level of infection protection. Regarding infection protection, EFSA stated that both direct and indirect contact with wild boars and hares must be avoided. Movements of infected live animals and semen constitute the main risk factors for the spread of infection in domestic pig production. Therefore, attention in the pig industry should be on detecting the clinical symptoms of brucellosis and on not feeding pigs with food waste, which is already prohibited in the EU [12].

If *B. suis* biovar 2 is introduced into a naïve domestic pig population, the clinical symptoms will likely be pronounced [20], but the response might depend on the dosages that the pigs are exposed to. Because exposure to *B. suis* may not necessarily result in initiating infection as pointed out by EFSA [12]. I in infected boars, the cardinal finding will be enlarged testicles.

The aim of this study was to undertake an assessment of the risk for human health associated with *B. suis* biovar 2 in pigs in Denmark. This was judged to have importance for all countries with a pig population, as the number of male pigs not subjected to surgical castration was expected to be on the increase, not just in Denmark but also elsewhere.

## Materials and methods

Relevant national and international legislation about pig health and meat inspection was consulted as well as literature and reports about *Brucella* spp. from EFSA. Expert knowledge was also retrieved from pig abattoirs in- and outside Denmark.

The risk assessment methodology used was developed by the WOAH. It consists of the following elements: (1) hazard identification, (2) release assessment, (3) exposure assessment, (4) consequence assessment and (5) risk estimation [21]. The hazard was a priori identified as *B. suis* biovar 2 in pigs based on a discussion among the coauthors of this paper. The release assessment described the biological pathways necessary for introducing *B. suis* biovar 2 to pigs and estimated the probability of this occurring. The exposure assessment described the biological pathways necessary for exposure of humans to *B. suis* biovar 2, here divided into human contact with live pigs (pig producers, pig veterinarians and abattoir workers) and the meat-borne route (consumers), and to estimate the probability of each of these events. The consequence assessment described the relationship between specified routes of exposure and the consequences of those exposures (here adverse health effects in humans) and estimated the probability of these events occurring. Finally, for risk estimation, the results from the release, exposure and consequence assessment were integrated to produce an overall assessment of the risk associated with *B. suis* biovar 2 in pigs, including the combined probability of the full chain of events occurring as well as the degree of consequences [21].

A semi-quantitative approach was chosen using an ordinal scale with five levels, i.e., negligible, very low, low, medium and high (Table 1). The time in years until the event was calculated to describe the probabilities of either a pig being infected (release assessment), a human being infected (exposure assessment) or an infected human falling ill (consequence assessment) in Denmark. Moreover, the uncertainty of the individual probability estimate was assessed using a qualitative scale with three levels, i.e., low, medium and high. The assessment “low uncertainty” designated probabilities where quantitative data were available, whereas “medium uncertainty” designated probabilities that were based on available information without quantitative data or where underreporting could not be excluded. Finally, the assessment “high uncertainty” designated probabilities where there was a scarcity of information.

**Table 1:** Definition of the scale used to describe the probability of a pig (release assessment) or a human (exposure assessment) being infected with a hazard of interest, or a human suffering from infection (consequence assessment) with the hazard of interest in Denmark^a^

Probability of occurrence	Wording
Negligible	Less than 1 case in 25 years
Very low	1 case in 10.1 to 25 years
Low	1 case in 5.1 to 10 years
Medium	1 case in 1.1 to 5 years
High	1 case or more within 1 year

^a^ Denmark has a population of 6 million people

The Danish pig population was divided into two: (1) indoors under controlled housing conditions and (2) outdoors or indoors under non-controlled housing conditions. Controlled housing demands high biosecurity. The specific requirements for a pig herd to be considered as a controlled housing farm are described in detail in the EU *Trichinella* Regulation [22]. In Denmark, pig farm compliance with controlled housing requirements is audited through a private standard called the DANISH Product Standard. Almost all Danish pigs are raised on farms that are part of this standard and most of the finishing pigs are from farms officially considered as applying controlled housing conditions [7]. In 2022, there were 2,576 active pig farms in total in Denmark, and 468 of these farms raised pigs outdoors, including organic farms and farms holding fenced-in wild boars [23, 24]. Outdoor pig farms are in general much smaller than indoor pig farms. Therefore, only a low proportion of pigs is raised outdoors: For more information on the DANISH Product Standard, please see: https://pigresearchcentre.dk/DANISH-quality-assurance-scheme/The-Danish-Product-Standard - link accessed 29 July 2025.

## Results

### Release assessment

#### Prevalence of Brucella suis biovar 2 in Denmark

Some EU Member States are apparently free of *B. suis* or report only sporadic outbreaks, while others report an increasing incidence [25]. In most countries, the wild boar maintains the infection with *B. suis* in wildlife, from which there is an occasional spill-over to other animals, such as cattle and pigs raised outdoors [16, 22]. Swine brucellosis disappeared in France in the 1970s with the industrialisation of pig production [26]. However, the infection is back again in some geographical areas, due to the establishment of outdoor pig production in areas where free-ranging wild boars are on the increase [25]. In fact, a total of 26 outdoor pig herds were detected as infected in France between 1993 and 2001 [26]. Likewise, 22 outdoor pig farms were detected in Germany between 2003 and 2023 [27].

Denmark does not have a free-living population of wild boar [28]. Instead, the European brown hares are thought to maintain the infection [10]. In 2002, two free-living European brown hares were detected as infected with *B. suis* biovar 2. The two hares were found dead in the region Himmerland, which is in the northern part of the Jutland peninsula. In 1994 and 1999, infection with *B. suis* biovar 2 was found in two outdoor pig herds in the same geographical area, where the pig veterinarian was called due to clinical manifestations. The source of the infection was never found, so it was assumed that the infection had been transmitted by infected hares living freely in the area [10]. The symptoms in the infected pigs were enlarged testicles in the boars and abortion in pregnant sows, which are the classic signs of infection with *Brucella* spp. Moreover, in 1973, *B. suis* biovar 2 was detected in a cow, also in the Himmerland region [29]. Trials undertaken in Denmark in the 1930 and 1950 involving heifers experimentally infected with *B. suis* indicated a low degree of pathogenicity [29].

No surveillance for *Brucella* spp. takes place in the Danish breeding pig herds. Instead, all boars are tested for *Brucella* spp. upon arrival to quarantine facility and before being moved to the boar stud as well as regularly when in the boar stud in accordance with EU Regulation 2020/688 and EU Directive 90/429/EEC12. Additionally, boars destined for export to certain countries outside the EU are tested. Due to the variation in numbers of exported boars, the number of tested boars differs substantially from year to year; during the period 2019–2023, the annual number of tested boar samples varied between around 20,000 and 43,000 [10, 30].

Each year, a few suspect cases of brucellosis are reported to the Danish Veterinary and Food Administration and are subsequently investigated. In 2021, this involved four pigs, a young bull and a ram. All six cases were detected on-farm in their respective herds. Three of the pigs were reported because they had reacted in a serological test. The three other animals were reported due to clinical appearance. Official restrictions were imposed on all involved herds until a negative confirmatory laboratory investigation had been made at the national reference laboratory, and all samples tested negative [30].

Cross-reactions between *Brucella* spp. and *Yersinia enterocolitica* O:9 are well-known in serological tests [12, 31]. This has special implication for breeding herds infected with *Y. enterocolitica* O:9, because boars from such herds can react positively for *Brucella* spp. on testing in connection with entrance to a boar stud. Hereby, the breeding herd of origin is subjected to official supervision until investigations have been made to reveal whether the infection is present or not [31].

#### Disease transmission and symptoms of brucellosis in pigs

According to EFSA, *B. suis* is most often transmitted from infected wild boars to outdoor domestic pigs via mating [12]. After introduction of the organism to a naïve domestic pig herd, reproductive symptoms will usually be seen. It is, therefore, a herd health issue, where diagnostics are central if the relevant symptoms appear [20].

The infection can be spread to the other pigs in the herd through consumption of feed or water contaminated with foetus, placenta, foetal fluids or vaginal discharge from infected sows, or via consumption of dead foetus or amniotic membrane [20]. Infected pigs can excrete *Brucella* in milk, urine and semen. Both symptomatic and asymptomatic boars can excrete *Brucella*, and transmission of infection during mating is thought to be common in pigs. Piglets can become infected in-utero or when they suckle the sow. Some of these young animals can become sero-negative carriers [20].

The clinical signs of brucellosis in pigs include abortions, stillbirths, the birth of weak piglets (which may die early in life) and small litter size. Although miscarriages can occur at any time during pregnancy, they are most common in mid- to late pregnancy. Abortions can be mistaken for infertility, because the sow or other pigs have eaten the aborted foetuses, and vaginal discharge is often minimal. Some abortion cases can be complicated by retention of afterbirth and secondary metritis. Epididymitis and orchitis are sometimes seen in boars and can result in infertility. Pigs that are not pregnant can have an asymptomatic course, although they can sometimes show lameness. Chronically infected herds show subtle signs, such as non-specific infertility, a slightly reduced farrowing frequency and irregular oestrous cycles. Deaths are rare except in foetal or newborn pigs [20].

#### Differential diagnosis

As with all diseases, it is important to be aware of the differential diagnoses. Finishing pigs in Denmark are usually around 5.5 months old when they are slaughtered. This implies that they are close to being sexually mature. At this age, the testicles of healthy, intact male finishing pigs are elliptic in shape, approximately 10 cm long, 6 cm wide and weigh 400 g per testicle (F. Keller, pig practitioner, personal communication, July 2025).

**Fig. 1:**
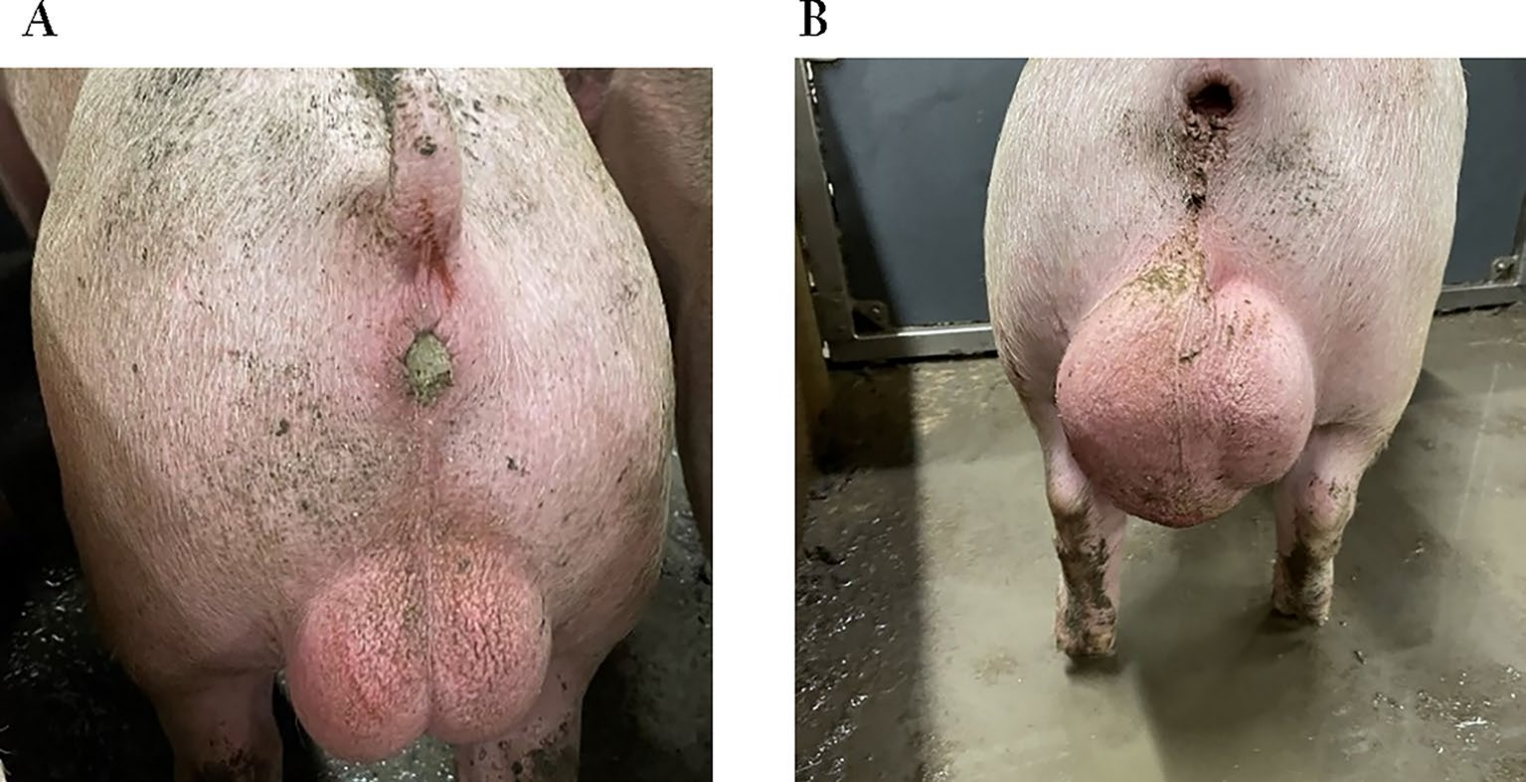
Two images showing an intact male pig with normal-appearing testicles to the left (Fig. 1A), whereas the intact male pig to the right had an asymmetric scrotum due to a greatly enlarged left testicle (Fig. 1B). The age of both pigs was approximately 5.5 months. The pig in Fig. 1B was suspected to be suffering from an inguinal hernia, based on a live examination Photo: Danish Veterinary and Food Administration. The inside of the scrotum is seen in Fig. S1A and B

As stated above, one of the cardinal symptoms of brucellosis is swollen testicles, which results in an increased size of the scrotum. However, the scrotum in male pigs can also be of increased size due to testicle torsion, traumatic injuries or hernia. Figure 1A shows a photo of an intact male pig with normally sized testicles, whereas Fig. 1B show an example of an intact male pig with enlarged testicles. Subsequent investigations showed that the enlargement was caused by multiple torsions of the testicle, which had resulted in stasis and ischaemia. The inside of the scrotum of the pig in Fig. 1B is shown in Figure S1A and Figure S1B. Castration can result in bleeding and subsequent development of adherences, but it is doubtful that this would manifest itself in the form of enlarged testicles at the time of slaughter. The porcine reproductive and respiratory syndrome (PRRS) virus is also associated with reproductive failures including abortions, but enlarged testicles is not a part of the clinical picture in boars [32]. Hence, in case of abortions where brucellosis cannot be ruled out, proper diagnostics are needed, including blood samples of live animals and/or necropsy after euthanasia or slaughter.

#### Conclusion regarding probability of release

The data used to assess the probability of release of *B. suis* biovar 2 in Danish pigs are presented in Table 2. The probability was assessed as negligible in live pigs raised indoors in Denmark, under controlled housing conditions. This implies a probability of less than one case in 25 years, corresponding to negligible probability. This estimate was assessed as having low uncertainty. Moreover, for live pigs raised outdoors, the probability was initially assessed as very low corresponding to one case in a time interval of more than 10 years and less than 25 years. This estimate was assessed as having a medium uncertainty, because the mild symptoms could mean that not all cases of *B. suis* biovar 2 infections would be detected in outdoor pig production (underreporting). Moreover, considering that symptoms may only cover abortions in sows and not swollen testicles in boar, pig producers and pig vets might consider PRRS virus and not *B. suis* biovar 2, the initial prevalence estimate for all outdoor-raised pigs, irrespective of location, was adjusted to from very low to low. Hence, most Danish pigs are not at risk of being exposed to infection with *B. suis* biovar 2. If infection enters a naïve pig herd, it will most likely be an outdoor herd. All outdoor-raised pigs, irrespective of location, were considered as having a low risk of *B. suis* biovar 2.

**Table 2:** Data used to estimate the probability of release of *B. suis* in Danish pigs

Country	Type of finding	Finding	Reference
France	Positive findings in livestock	Twenty-six outdoor pig herds between 1993 and 2001	Mailles et al., 2017 [26]
Denmark	Positive findings in wildlife and livestock	Two hares in 2002 One outdoor pig farm in 1994 and one outdoor pig farm in 1999 One cow in 1973	Danish Veterinary and Food Administration, 2024 [10] Andersen & Pedersen, 1995 [29]
Denmark	Surveillance in boars for export	Between approximately 20,000 and 43,000 tests were made annually between 2019 and 2023 – no positives were found	Danish Veterinary and Food Administration, 2023 & 2024 [10, 30]

### Exposure assessment

According to EFSA, humans can be exposed to *Brucella* spp. through handling infected live animals [12]. Pig producers’ and pig veterinarians’ contact with the placenta from infected animals is an example of such a route. Another somewhat similar exposure route is through inhalation of bacteria in aerosols generated following abattoir workers’ dressing of an infected carcass [26]. Additionally, exposure can occur via consumption of *Brucella*-contaminated milk, cheese and other dairy products that have not undergone pasteurisation. Consumption of meat is not believed to be an exposure route unless the meat originates from acutely infected animals [8, 12].

In 2002, a legislative requirement was introduced in France specifying heat treatment of meat from animals infected with *Brucella* to a core temperature of at least 65 °C. This requirement was introduced after – as described above in Sect. "Release assessment" – outbreaks of *B. suis* biovar 2 in 26 outdoor pig herds in France [33]. The requirement was introduced as a precaution against the potentially zoonotic nature of *B. suis*. In 2005, the need for this risk mitigation was investigated in a study on behalf of the French authorities [33]. The study showed that none of the pig producers involved in the *B. suis* outbreaks in outdoor-reared pigs, or their families, reported human cases of brucellosis or related symptoms. The conclusion was that there was no need for heat treatment of the meat, as the pathogenicity was assessed as low, whereas condemnation of the offal was considered appropriate. According to the authors, a heat treatment requirement could still be necessary for outbreaks involving biovars other than biovar 2 [33].

EU Regulation 2019/627 on official controls on products of animal origin intended for human consumption specifies, in paragraph 34, the following practical arrangements for official controls regarding brucellosis during PM inspection [8]:

1. If animals have reacted positively or inconclusively to a brucellosis test, or there are other reasons to suspect infection, they must be slaughtered separately from other animals, taking precautions to avoid the risk of contamination of other carcasses, the slaughter line and the staff present in the abattoir.
2. Meat from animals in which PM inspection has shown changes suggestive of acute brucellosis shall be declared unfit for human consumption. In the case of animals which react positively or inconclusively to a brucellosis test, the udder, genitals and blood shall be declared unfit for human consumption, even if no changes have been found.

The view that meat is a safe commodity in relation to *Brucella* is also shared by WOAH [11]. This implies that when authorising import or transit of meat, veterinary authorities should not require any *Brucella*-related conditions, regardless of the *Brucella* infection status of the animal population of the exporting country.

#### Conclusion regarding probability of exposure

*Brucella* is primarily transmitted to humans through contact with infected animals, their excretions, e.g., milk and milk products, and their tissues, e.g., amniotic membranes. The EU legislation distinguishes between acute and chronic brucellosis in livestock, where acutely infected livestock is euthanized and condemned, while in chronic conditions, only organs and tissues associated with infection are condemned. This is because meat from chronically infected animals does not pose a risk of infection to humans. The probability of human exposure due to the meat-borne route was assessed as negligible, implying less than one case in 25 years in Denmark. The uncertainty was judged as medium. Likewise, the probability of exposure due to contact was assessed as low, implying one case in 5.1 to 10 years in Denmark, if infection would be present in pigs. Importantly, the pig producer and the practicing veterinarian must never forget *B. suis*, whenever reproductive problems are observed in an outdoor herd. The uncertainty was judged as medium.

### Consequence assessment

In humans, the onset of brucellosis can be insidious or abrupt. Acute brucellosis is usually a febrile illness with nonspecific flu-like symptoms such as fever, chills, headache, malaise, back pain, myalgia and lymphadenopathy, which can be accompanied by splenomegaly and/or hepatomegaly. Patients can experience profuse sweating, especially at night. Nonspecific gastrointestinal signs, including anorexia, vomiting, diarrhoea and constipation, can also be seen. Some individuals recover spontaneously, while others develop persistent nonspecific symptoms, including undulant fever [20].

According to EFSA [12] and Vaillant et al. [33], *B. suis* biovar 2 is rarely pathogenic for humans, whereas biovars 1 and 3 are highly pathogenic and can cause serious disease. Only a few human cases of biovar 2 have been described in the literature. One of the well-described cases occurred in 1989, and involved a 20-year-old French farmer, where the cause of infection could not be found [34]. Later, as described below, a total of seven human cases of brucellosis were detected in France between 2004 and 2016 [26]. They were diagnosed in each their region in mainland France, and none of them were related. All seven had previously been in contact with wild boar while hunting or preparing wild boar meat. None of them had a history of exposure to domestic pigs. The median age of the seven cases was 68 years. Five of the seven had comorbidities in the form of chronic diseases and/or suffered from immunosuppressive conditions [26].

The mild symptoms could indicate that *B. suis* biovar 2 infections in humans are underdiagnosed [20, 26]. Nonetheless, cases of disease in humans caused by biovar 2 have also been seen, where the symptoms were like the clinical conditions seen in brucellosis caused by other *Brucella* biovars [26].

In Denmark, between one and seven human cases of *B. abortus* or *B. melitensis* were detected annually between 2018 and 2023. Most have been cases related to travel abroad or travellers bringing infected food home [35]. No human cases of *B. suis* have ever been detected in Denmark.

#### Conclusion regarding probability of consequences

If humans are exposed to *B. suis* biovar 2, no matter whether it is through the meat-borne route or due to contact, the probability of developing serious disease was assessed as low, because of the low pathogenicity observed, implying one case in 5.1 to 10 years in Denmark, if exposure would happen. The uncertainty was judged as medium.

### Risk estimate

*Brucella suis* was identified as the only relevant public health hazard in Denmark in relation to not inspecting the testicles of male pigs. The risk associated with a person becoming ill due to exposure to *B.* suis biovar 2 in Danish pigs was calculated as the total probability of the event happening and is shown in Table 3, which also contains a summary of the estimated probabilities for release, exposure and consequence, divided according to route of exposure (meat-borne or contact) and type of pig production (indoor or outdoor). It is notable that for indoor production, the risk was assessed as negligible for both the meat-borne route and the contact route, because *B. suis* has not been found in indoor pigs for more than 50 years. For outdoor-raised pigs, the risk was estimated as low for the contact route, whereas the risk associated with the meat-borne route was assessed as negligible because *B. suis* is not considered as meat-borne, unless the meat originates from animals that are acutely infected, and such animals are not allowed to be slaughtered.

**Table 3:** Estimates of the individual probabilities of release, exposure, consequences and the derived total risk of humans falling ill due to *Brucella suis* in Danish pigs, divided according to route of exposure and type of pig production

Pig system	Exposure route	Release	Exposure	Consequences	Risk estimate
Outdoor	Contact	Low	Low	Low	Low
Outdoor	Meat-borne		Negligible		Negligible
Indoor	Contact	Negligible	Low		
Indoor	Meat-borne		Negligible		

## Discussion

*Brucella suis* was identified as the only relevant public health hazard in relation to not detecting abnormal testicles of male finishing pigs. Our risk assessment showed that the probability of a person in Denmark falling ill from *B. suis* is very low to negligible, because the infection has only been observed on a few occasions over the last 50 years and never in humans. The few findings have been in either wildlife or outdoor reared livestock. If infection should enter a naïve, outdoor pig herd, it can spread rapidly and result in macroscopically recognizable symptoms, such as increased size of testicles [12]. Moreover, abortions in larger numbers will occur, and this will likely be noted and reacted upon, because pig farmers monitor the reproduction of their sows routinely due to the economic implications. Hence, unnoticed on-farm infections are not that likely, although some underreporting cannot be excluded if only abortions are observed leading to focus on PRRS virus when submitting samples for diagnosis. Pig producers and people working in outdoor pig farms could in theory become infected prior to detection of the infection, but the probability is likely low as shown in the French study where none of the seven human cases were related to pig production [33]. Next, the meat from infected pigs is not considered infectious, and the infection is not considered as being associated with severe symptoms, unless the person exposed has comorbidities.

The lack of human cases in Denmark made it a challenge to calculate the individual probabilities of exposure and consequences specifically for Denmark. However, detailed data from France and EFSA were available [12, 26, 33], whereby estimates could be derived, pointing to a low or negligible total risk, depending on the route of exposure and population of interest.

Still, it is an EU requirement that all pigs, regardless of way they are raised, undergo ante-mortem (AM) inspection before slaughter, and only pigs that are accepted at AM inspection can be slaughtered and subjected to PM inspection. As explained above, the concept of controlled housing is used in Denmark to distinguish between pigs raised indoors under high biosecurity and all other pigs as described in the EU *Trichinella* Regulation [22]. Prior to sending a batch of pigs for slaughter, the pig producer must inform the abattoir of the way the pigs were raised, indoors or outdoors, as part of the food chain information [7]. At the large, export-authorised abattoirs which slaughter most of the Danish pigs, two different methods of PM inspection are in place. Pigs raised under controlled housing conditions are subjected to visual-only PM inspection, unless serious findings are made during inspection, whereas all other pigs are subjected to the traditional inspection, involving incisions and palpations [7].

The risk related to *B. suis* biovar 2 is likely negligible in indoor pig farms, not just in Denmark, but in all EU Member States, because the same kind of biosecurity and risk mitigating measures, including housing conditions and feeding systems, are applied which prevents transmission of infection with *B. suis* from wild animals. Moreover, only pigs from farms applying equivalent biosecurity and risk mitigating measures are being introduced. Therefore, we propose a simplification of the EU Regulation 2019/627, whereby testicles could be handled as the penis and discarded by the Food Business Operator before the PM inspection if the pigs are raised indoors (or under controlled housing conditions) in an EU Member state that is officially free from *B. abortus* and *B. melitensis*. This might initially seem problematic in EU Member States that are free from *B. suis* biovar 2, where there is no routine surveillance on-farm, because official inspection at the abattoir might be perceived as the only remaining instrument for disease surveillance and, therefore, removing any aspect of that may undermine capacity for detection. However, according to Article 19 and Annex 3 in the EU Regulation 2020/688, the focus should be on outdoor-raised pigs, and it is mandatory to have one annual farm visit by a veterinarian [19]. Moreover, official inspection of the testicles of the intact males and immuno-castrated males from outdoors (or non-controlled housing) should continue, because these animals represent the highest risk of *B. suis*. Hereby, they act as sentinels in a national surveillance. As discussed for bovine tuberculosis by Foddai et al. [36], it is the agricultural structure and not the surveillance system represented by PM inspection at the abattoir, which is of primary relevance when estimating the probability of freedom from disease in a presumably disease-free country. Moreover, a distinction between indoor and outdoor (or controlled housing versus non-controlled housing) would be in line with EFSA’s recommendations regarding use of harmonised epidemiological indicators, where finisher pigs from farms officially recognised as applying controlled housing conditions were considered as low risk for *Trichinella* spp. and *Toxoplasma gondii*, two hazards that are also more commonly found outdoors than indoors, and therefore, auditing of the farm biosecurity would suffice [37]. For *Trichinella*, this is today allowed in the EU [22]. In this context, it would be interesting to have EFSA’s judgement regarding:

1. The value of PM inspection of the testicles of indoor raised, intact male pigs for disease detection while continuing with PM inspection of the testicles of the outdoor raised intact male pigs and.
2. The impact of omitting this inspection of indoor raised intact male pigs, under the conditions described above, on the EU animal health rules.

Moreover, a computer vision system could be developed that could inspect and detect enlarged testicles on carcass of intact male pigs at the abattoir. Hereby, the carcasses with enlarged testicles could be sent directly to the rework area for a detailed follow-up by official inspection. Carcasses with normal sized testicles could be handled by the food business operator. This would fit with the legal instructions in force in Denmark, where presence of orchitis and epididymitis would demand inspection from the competent authority and result in local condemnation only, unless complications are seen [38]. Complications could e.g. consist of lesions indicating systemic involvement. Both suggestions would require amendments to the EU legislation. However, with the speed of development of computer-based vision systems [39, 40], it is more a question of when than whether such technologies will form part of a modern and robust meat inspection in the future.

Finally, the conclusions of this risk assessment can be used in case one testicle is missing at the PM inspection, whereby the EU legislation’s requirement for inspection of all parts of a slaughtered animal is not complied with. On the condition that the remaining testicle looks normal, then there is a negligible probability that the animal was infected with *B. suis*, if the case deals with a male pig that has been raised indoors. If the case involves an outdoor-raised male pig, then the probability that the animal was infected with *B. suis* is very low, because although the infection could be present, it is unlikely that only the missing part of the testicles is showing symptoms represented by being swollen, as brucellosis is a systemic infection.

## Conclusion

Control of *Brucella suis* biovar 2 has international interest despite that the infection is rarely observed within pig production and the pathogenicity is low. The risk for a person in Denmark related to *B. suis* biovar 2 was assessed as low in case of contact with outdoor-reared pigs and negligible for the meat-borne route and contact to indoor raised pigs. This was mainly because the infection has only been observed on a few occasions in Denmark over the last 50 years; namely one cow, two outdoor pig farms and two hares, and never in humans. Hence, it is not the risk of brucellosis that entails the need for official inspection of the testicles of intact male or immuno-castrated pigs, reared indoors in Denmark, but the EU requirement for PM inspection of all parts of the carcass and organs except the penis. Male pigs with enlarged testicles, found on-farm or during AM or PM inspection, should always be examined in-depth, irrespective of whether the pig is alive or slaughtered, and raised indoors or outdoors. Such examination should include euthanasia of the pig and subsequent incision into the scrotum to determine whether the condition is caused by a testicular torsion or scrotal/inguinal hernia, which constitute the differential diagnoses. If these are not the causes, further laboratory work must be undertaken to determine the reason behind the swelling.

## Epilogue

On 26 August 2025, it was officially announced that *B. suis* biovar 2 had been detected in an outdoor pig farm in Denmark. The finding has been notified to WOAH.

## Supplementary Information

Below is the link to the electronic supplementary material.

Supplementary Material 1

## Data Availability

Data are available upon contact to the first author.

## References

[1] Font-i-Furnols M, Aaslyng MD, Backus GBC, Han J, Kuznetsova TG, Panella-Riera N, et al. Russian and Chinese consumers’ acceptability of Boar meat patties depending on their sensitivity to Androstenone and Skatole. Meat Sci. 2016;121:96–103. 27294519 10.1016/j.meatsci.2016.06.003

[2] Bonneau M, Weiler U. Pros and cons of alternatives to piglet castration: Welfare, Boar Taint, and other meat quality traits. Animals. 2019;9:884. 10.3390/ani9110884. 31671665 10.3390/ani9110884 PMC6912452

[3] Morales J, Dereu A, Manso A, De Frutos L, Piñeiro C, Manzanilla EG, et al. Surgical castration with pain relief affects the health and productive performance of pigs in the suckling period. Porcine Health Manag. 2017;3:18. 10.1186/s40813-017-0066-1. 28879020 10.1186/s40813-017-0066-1 PMC5585944

[4] Maribo H, Nielsen MBF, Krustrup AK. Productivity and boar taint in castrates, male pigs and immunocastrates. Trial Report No. 1219. SEGES – Danish Pig Research Centre. 2021:19.

[5] Shtepliuk I, Domènech-Gil G, Almqvist V, Kautto AH, Vågsholm I, Boqvist S, et al. Electronic nose and machine learning for modern meat inspection. J Big Data. 2025;12:96. 10.1186/s40537-025-01151-4.

[6] Dunshea FR, Colantoni C, Howard K, McCauley I, Jackson P, Long KA et al. Vaccination of boars with a GnRH vaccine (IMPROVAC) eliminates boar taint and increases growth performance. J Anim Sci 2001;79:2524-35. 10.2527/2001.79102524x 10.2527/2001.79102524x 11721830

[7] Alban L, Enemark HL, Petersen HH, Nielsen LH. Auditing of Danish pig herds for controlled housing requirements and Trichinella. Food Waterborne Parasit. 2024;37:e00247. 10.1016/j.fawpar.2024.e00247. 10.1016/j.fawpar.2024.e00247 PMC11483480 39421688

[8] EU Commission. EU regulation 2019/627 laying down uniform practical arrangements for the performance of official controls on products of animal origin intended for human consumption. 2019. https://eur-lex.europa.eu/legal-content/EN/TXT/PDF/?uri=CELEX:32019R0627.

[9] EU Parliament and Council. EU regulation 853/2004 laying down specific hygiene rules for on the hygiene of foodstuffs. 2004. https://eur-lex.europa.eu/legal-content/EN/TXT/PDF/?uri=CELEX:32004R0853

[10] Danish Veterinary and Food Administration. Animal Health in Denmark 2023. 2024 Animal Health in Denmark 2023.

[11] WOAH. Chapter 8.4. Infection with Brucella abortus, B. melitensis and B. suis. Terrestrial Animal Health Code. 2025a https://www.woah.org/fileadmin/Home/eng/Health_standards/tahc/2018/en_chapitre_bovine_brucellosis.htm

[12] EFSA. Scientific opinion of the panel on Animal Health and Welfare (AHAW) on a request from the Commission on porcine brucellosis (Brucella suis). Porcine brucellosis (Brucella suis) - – 2009 - EFSA Journal - Wiley Online Library. EFSA Journal. 2009;1144:1-112.

[13] Pinn-Woodcock T, Elisha Frye E, Guarino C, Franklin-Guild R, Newman AP, Bennett J, et al. A one-health review on brucellosis in the united States. J Amer Vet Med Assoc. 2023;261:4. 10.2460/javma.23.01.0033. 10.2460/javma.23.01.0033 36862545

[14] Tomaselli M, Elkin B, Kutz S, Harms NJ, Nymo HI, Davison T, et al. A transdisciplinary approach to Brucella in muskoxen of the Western Canadian Arctic 1989–2016. EcoHealth. 2019;16:488–501. 10.1007/s10393-019-01433-3. 31414318 10.1007/s10393-019-01433-3 PMC6858907

[15] Kneipp CC, Sawfords K, Wingett K, Malik R, Stevenson MA, Mor SM, et al. *Brucella suis* Seroprevalence and associated risk factors in dogs in Eastern Australia, 2016 to 2019. Front Vet Sci. 2021;8:727641. 10.3389/fvets.2021.727641. 34621810 10.3389/fvets.2021.727641 PMC8490753

[16] Brangsch H, Horstkotte MA, Melzer F. Genotypic peculiarities of a human brucellosis case caused by *Brucella suis* biovar 5. Sci Rep. 2023;13:16586. 10.1038/s41598-023-43570-4. 37789135 10.1038/s41598-023-43570-4 PMC10547717

[17] Anonymous. Annual Report on Zoonoses in Denmark 2023. National Food Institute, Technical University of Denmark. 2024 https://www.zoonoses_annual_report_2023_final_.pdf.

[18] EU Commission. EU Regulation 2018/1882 on the application of certain disease prevention and control rules to categories of listed diseases and establishing a list of species and groups of species posing a considerable risk for the spread of those listed diseases. 2018 https://eur-lex.europa.eu/legal-content/EN/TXT/PDF/?uri=CELEX:32018R1882

[19] EU Regulation 2020/688 supplementing EU Regulation 2016/429 of the European Parliament and of the Council, as regards animal health requirements for movements within the Union of terrestrial animals and hatching eggs. 2020 https://eur-lex.europa.eu/legal-content/EN/TXT/PDF/?uri=CELEX:32020R0688

[20] Anonymous. 2018. Fact sheet on *Brucella suis*. https://www.cfsph.iastate.edu/Factsheets/pdfs/brucellosis_suis.pdf

[21] WOAH. Handbook on Import Risk Analysis. Introduction and qualitative risk analysis. Second Edition. World organisation for animal health. 2010;1. https://www.handbook_on_import_risk_analysis_-_oie_-_vol__i.pdf.

[22] EU Commission. EU Regulation 2015/1375 laying down specific rules on official controls for Trichinella in meat. 2015 https://eur-lex.europa.eu/legal-content/EN/TXT/PDF/?uri=CELEX:32015R1375

[23] Danish A. & Food Council. Statistics for the Danish pig production. 2025. Available from: https://agricultureandfood.co.uk/news/statistics (accessed August 1, 2025).

[24] Gao Y, Nielsen LH, Boklund AE, de Jong MCM, Alban L. SWOT analysis of risk factors associated with introduction of African swine fever through vehicles returning after export of pigs. Front Vet Sci. 2023;9:1049940. 10.3389/fvets.2022.1049940. 36686159 10.3389/fvets.2022.1049940 PMC9846816

[25] Girault G, Djokic V, Petot-Bottin F, Perrot L, Thibaut B, Sébastien H, et al. Molecular investigations of two first Brucella suis biovar 2 infection cases in French dogs. Pathogens. 2023;12:792. 10.3390/pathogens12060792. 37375482 10.3390/pathogens12060792 PMC10304029

[26] Mailles A, Ogielska M, Kemiche F, Garin-Bastuji B, Brieu N, Burnusus Z, et al. *Brucella suis* biovar 2 infection in humans in france: emerging infection or better recognition? Epidemiol Infect. 2017;145:2711–6. 10.1017/S0950268817001704. 28784192 10.1017/S0950268817001704 PMC9148767

[27] Melzer F, Linde J, Brangsch H. Genomic epidemiology of *Brucella suis* biovar 2 in German swine and wildlife, 2003–2023. Front Vet Sci. 2025;12:1611681. 10.3389/fvets.2025.1611681. 40625701 10.3389/fvets.2025.1611681 PMC12231500

[28] Jordt AM, Lange M, Kramer-Schadt S, Nielsen LH, Nielsen SS, Thulke H-H, et al. Spatio-temporal modeling of the invasive potential of wild boar - a conflict-prone species - using multi-source citizen science data. Prev Vet Med. 2016;124:34–44. 10.1016/j.prevetmed.2015.12017. 26775815 10.1016/j.prevetmed.2015.12.017

[29] Andersen FM, Pedersen KB. Brucellosis – A case of natural infection with *Brucella suis* biotype 2 in a cow (in Danish). Dansk Veterinærtidsskrift. 1995;78(8):408.

[30] Danish Veterinary and Food Administration. Animal Health in Denmark 2022. 2023 Animal Health in Denmark 2022.

[31] Kristensen CS, Boes J, Enøe C, Jungersen G. New Brucella/Yersinia 0:9 diagnostic (in Danish). Message 650. 2014 https://svineproduktion.dk/publikationer/kilder/lu_medd/2004/650

[32] WOAH. Porcine reproductive and respiratory syndrome. 2025a Porcine reproductive and respiratory syndrome-WOAH-World Organisation for Animal Health Linked accessed 1 August 2025.

[33] Vaillant V, Garin-Bastuji B, Louguet Y, Brun M. Séroprévalence Humaine autour des foyers porcins de brucellose à Brucella suis biovar 2, France, 1993–2003. Inst Veill Sanit. 2005;1:44. https://www.santepubliquefrance.fr/content/download/185766/2316922.

[34] Teyssou R, Morvan J, Leleu JP, Roumegou P, Goullin B, Carteron B. A Propos d’un Cas de brucellose Humaine a *Brucella suis* biovar 2. Med Mal Infect. 1989;19(3):160–1. 10.1016/S0399-077X(89)80221-5.

[35] Statens Seruminstitut. Brucellose. 2024 Brucellose. Link accessed 1 August 2025.

[36] Foddai A, Nielsen LR, Willeberg P, Alban L. Comparison of output-based approaches used to substantiate bovine tuberculosis free status in Danish cattle herds. Prev Vet Med. 2015;121:21–9. 26036341 10.1016/j.prevetmed.2015.05.005

[37] EFSA. Scientific report on technical specifications on harmonised epidemiological indicators for public health hazards to be covered by meat inspection of swine. EFSA J. 2011;9(10):2371. 10.2903/j.efsa.2011.2371. [125 pp.] Technical specifications on harmonised epidemiological indicators for public health hazards to be covered by meat inspection of swine.

[38] Ministry of Food, Agriculture and Fisheries of Denmark. Guidance No. 9525 regarding the conduct of meat inspection (in Danish). 2025 https://www.retsinformation.dk/eli/retsinfo/2022/10380

[39] Lund DH, Alban L, Hansen C, Dalsgaard A, Denwood M, Olsen A. Using latent class modelling to evaluate the performance of a computer vision system for pig carcass contamination. Prev Vet Med. 2025a;241:106556. 40359587 10.1016/j.prevetmed.2025.106556

[40] Sandberg M, Ghidini S, Alban L, Dondona AC, Blagojevic B, Bouwknegt M, et al. Applications of computer vision systems for meat safety assurance in abattoirs: A systematic review. Food Control. 2023;150:109768. 10.1016/j.foodcont.2023.109768.

