# Supplementary material for: Risk assessment for Brucella suis biovar 2 in Danish pigs
Source: Porcine Health Manag. 2025 Nov 17;11:60. doi: 10.1186/s40813-025-00471-4 (PMC12625320; doi:10.1186/s40813-025-00471-4)
Supplement: Supplementary file 1 — Supplementary Material 1 [file 40813_2025_471_MOESM1_ESM.docx]

**Supplementary materials**

Figure 1A in the core paper shows a photo of an intact male pig with normally sized testicles, whereas Figure 1B show an example of an intact male pig with enlarged testicles. The pig in Figure 1B was transported separately to the abattoir, with a suspected hernia. According to the official veterinarian who received the pigs at slaughter, the area above the left testicle felt hardened and thickened on palpation. The left part of the scrotum was approximately three times larger than the right, which had a normal texture and was only slightly increased in size. The affected intact male did not show any indication of pain or discomfort during the clinical examination.

The inner side of the scrotum of the pig in Figure 1B is shown in Figure S1A and Figure S1B, including the inguinal channels and the testicles. Massive fibrosis is observed with blood and plasma exudation and fibrinous adherences on the inner side of the scrotum. The right inguinal channel was also oedematous, but to a lesser extent than the left. Figure S1B shows that the left testicle had been twisted several times, whereas the right testicle had a normal appearance with a mild degree of oedema. The multiple torsions of the testicle had resulted in stasis and ischaemia.


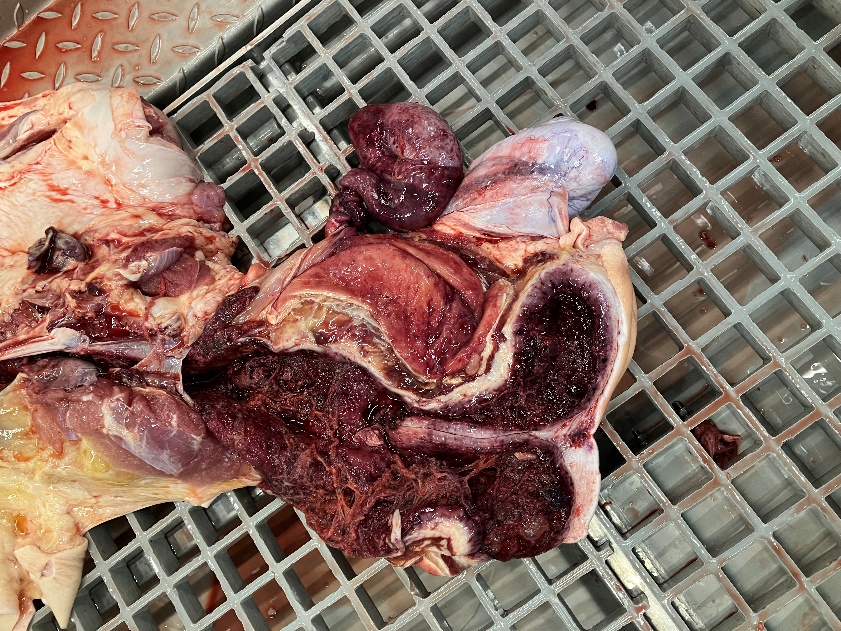

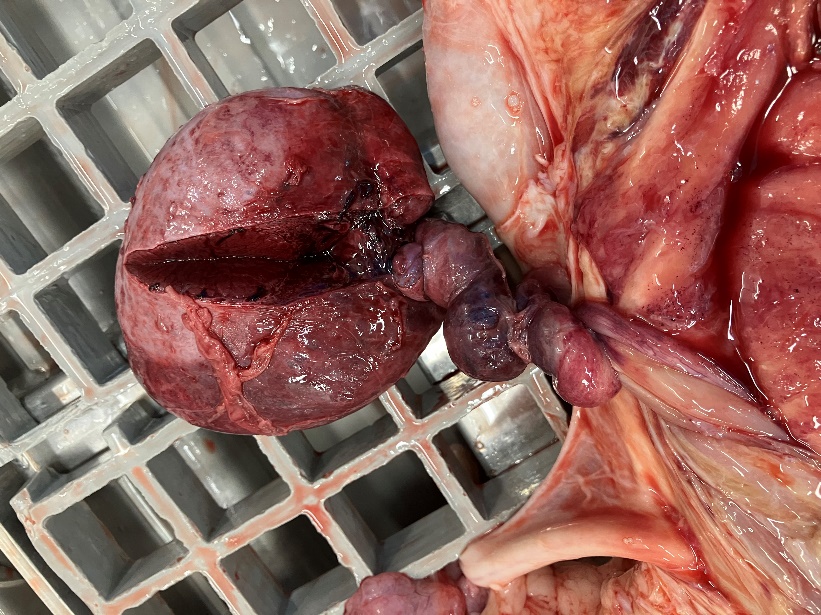


**Fig. S1** Two images showing the inside of the scrotum of a male pig, presented at slaughter with swollen testicles. As shown in Fig. S1A, the inguinal canal was filled with blood, plasma was leaking and there were fibrinous adhesions. Fig. S1B is a close-up of the left testicle, showing multiple torsions of the testicle with stasis and ischemia. Fig. 1B shows the scrotum of the same pig when a live. Source: Danish Veterinary and Food Administration.


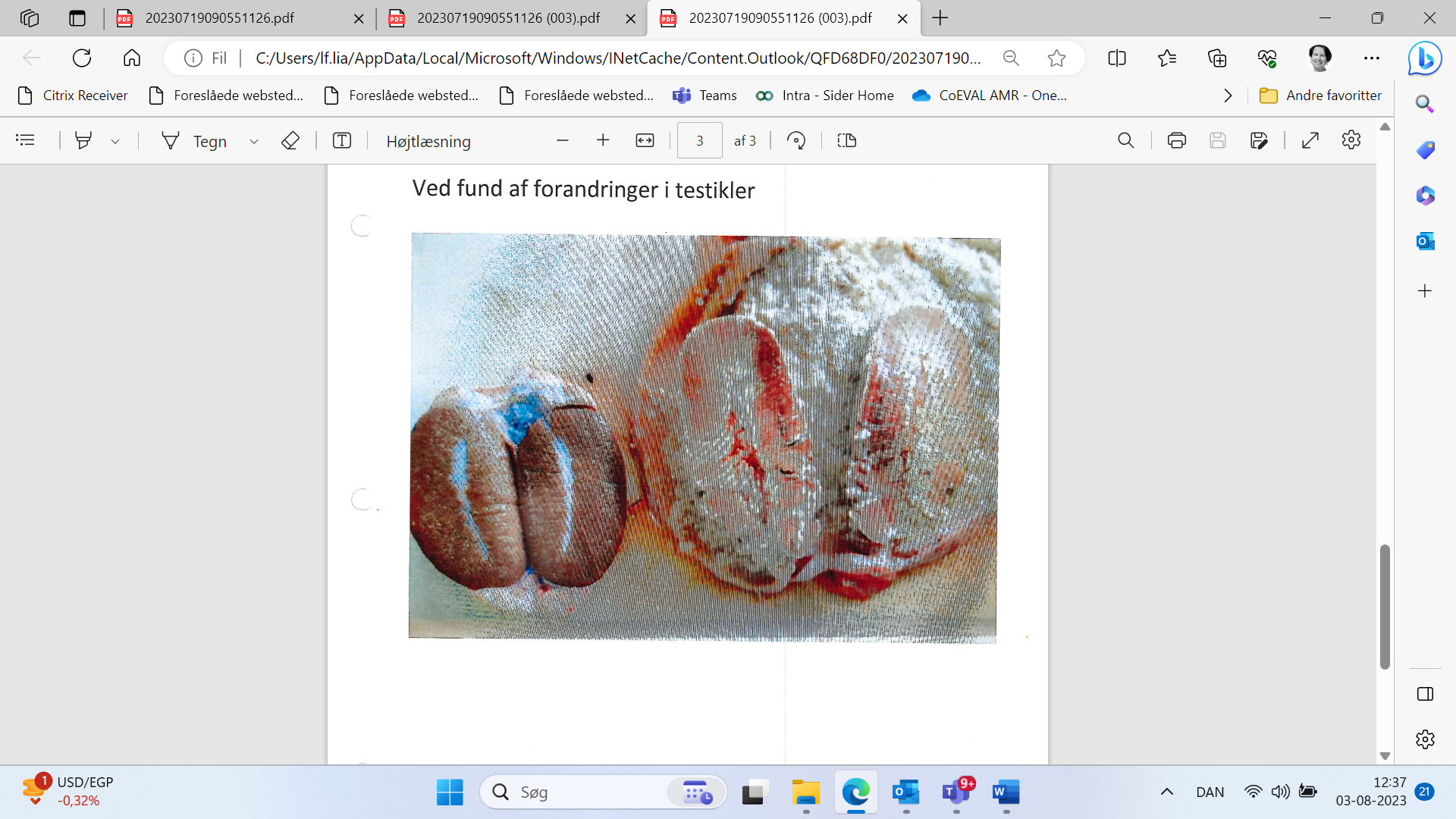


**Figure S2** – Photos of testicles of normal size (marked with blue, to the left) and testicles from a boar with brucellosis (marked with red, to the right). Source: Danish Veterinary and Food Administration.
